# Genomic landscapes of breast cancer in African populations: a systematic review

**DOI:** 10.1038/s41523-025-00809-9

**Published:** 2025-08-14

**Authors:** Nduta Mugo, Gianmarco Contino

**Affiliations:** 1 Department of Cancer and Genomic Sciences, College of Medicine and Health, University of Birmingham, Edgbaston, UK; 2 Upper Gastrointestinal Surgery, University Hospital of Birmingham NHS Trust, Birmingham, UK; 3 VHI Institute, University of Cambridge, Cambridge, UK

**Keywords:** Cancer, Genetics, Cancer genomics, Population genetics

## Abstract

Breast cancer in African women carries high mortality, yet genomic data from continental African cohorts are limited. We conducted a systematic review of next-generation sequencing based observational cohort studies of somatic breast cancer genomes in women of African ancestry, from January 2004 to September 2024. Extracted genomic features—driver mutations, copy-number alterations, structural-variants, tumour mutational burden and mutational signatures—were contrasted against TCGA’s Black/African American and White reference cohorts. Ten studies from seven countries met inclusion criteria. Main findings were higher rate of triple-negative breast cancer, higher rate of *TP53* and lower rate of *PIK3CA* mutations compared with TCGA White. African tumours also exhibited elevated TMB, CNA and SV burden. Mutational signatures revealed enriched homologous recombination deficiency and APOBEC activity. Despite small, heterogeneous cohorts and regional gaps, these distinct somatic landscapes suggest PARP, PI3K and immunotherapy targets. Coordinated sequencing efforts are urgently needed to drive precision oncology in Africa.

## Introduction

Breast cancer is a heterogeneous disease composed of distinct subtypes that include molecular pathology, histology, genetic and gene expression profiles. Subtyping has a strong impact on the prediction of response to clinical outcomes and treatment. Molecular pathology involves the immunochemical quantification of estrogen receptor (ER), progesterone receptor (PR) and HER2(ERBB2) expression^1^. Tumours that lack ER, PR and HER2 expression are termed as triple-negative breast cancer (TNBC). TNBCs are characterised by aggressive clinical courses and lack of targetable genomic alterations^2^. The higher incidence of triple-negative breast cancer among African American women and sub-Saharan Africa has raised questions on the role of African ancestry as a susceptibility factor for disease phenotypes^3^.

Female breast cancer (BC) is the most frequently diagnosed cancer and the fourth leading cause of cancer deaths worldwide with an estimated 2.3 million cases and 666 000 deaths reported in 2022^4^. Non-communicable diseases such as cancer, cardiovascular diseases, and type II diabetes are significantly altering the disease landscape in Africa^5^. According to GLOBOCAN 2022 data, breast cancer is the leading cause of cancer incidence and mortality of women in Africa at 40.5 and 19.2 per 100 000 respectively^4^. This cancer burden is expected to double by 2040^6^.

Somatic mutations^7^, which are genomic alterations acquired throughout an individual’s lifetime, are a universal feature of cancer genomes^8^. These mutations include various DNA changes such as base substitutions; insertions and deletions (indels), copy number variations that either amplify segments or completely remove them, and structural rearrangements where DNA segments break and reattach at new locations^8^. In contrast, germline mutations are inherited directly from parents^8^.

Driver mutations are a subset of somatic mutations that confer a selective growth advantage to tumour cells^9^, thus causally associating them with tumorigenesis^7^. Passenger mutations although not directly contributing to cancer development, can provide insight into the aetiology and progression of cancer^7^. Different landscapes of somatic mutations can be associated with the variances in the biology of cancer and response to treatment and prognosis. Somatic mutations can function as predictors of tumour response, which has subsequently led to the development of targeted cancer therapies^10^.

Africa, with over 2000 ethnolinguistic groups, presents exceptional genomic diversity. This diversity affords a unique lens through which to study somatic mutational process and oncogenic mechanisms. Comparative analyses suggest that somatic mutation profiles differ markedly across ancestries^11^, yet African populations remain substantially underrepresented in cancer genomic research. This systematic review aims to consolidate and critically appraise the current body of literature on somatic mutations in breast cancer, with a specific focus on women of African ancestry.

## Results

**Fig. 1:**
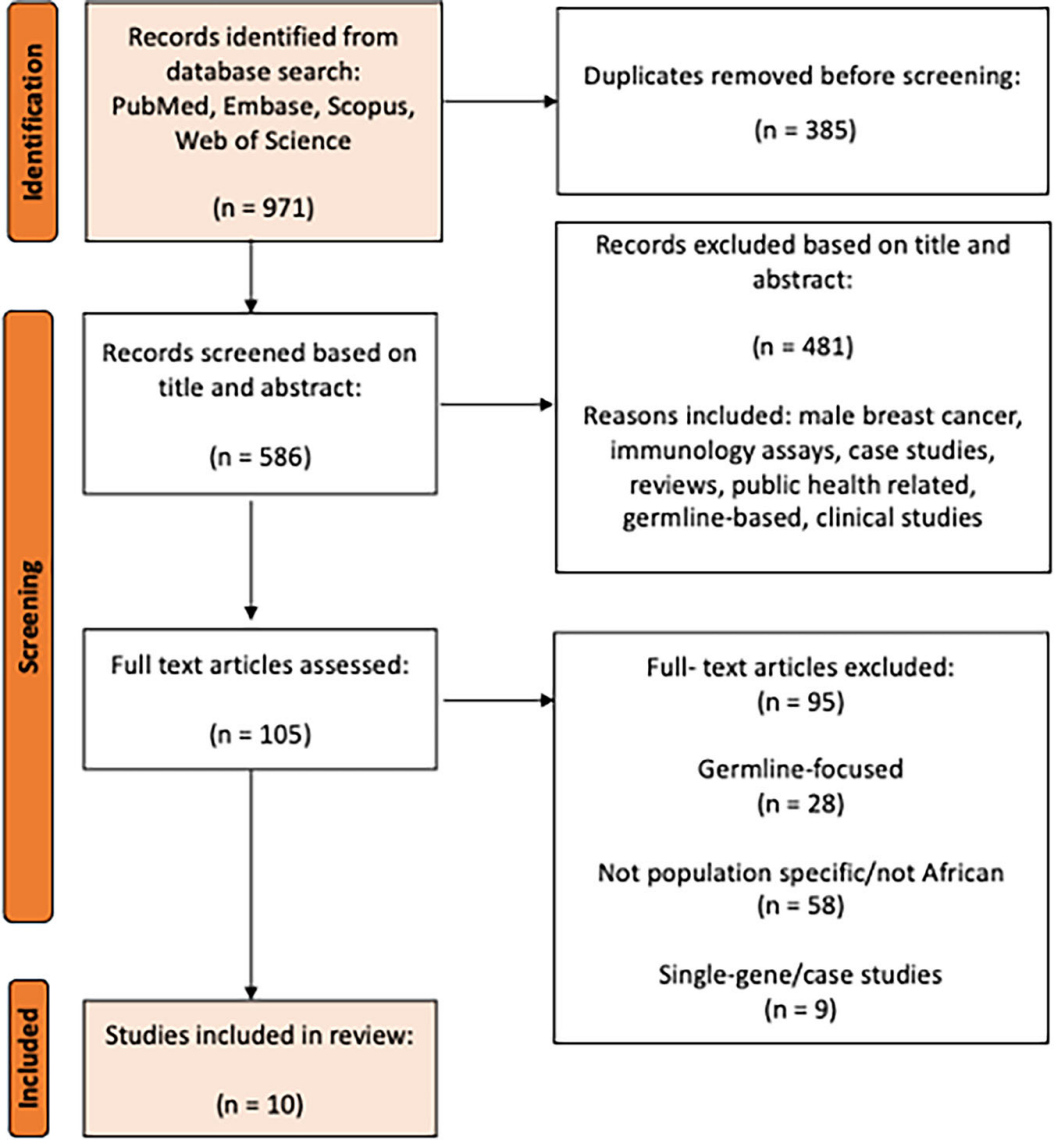
PRISMA flow chart of the literature search strategy.

A total of 971 publications were identified from searches conducted on PubMed, Scopus, Web of Science and Embase based on the selected search descriptors in each database (Supplementary Table 1a–d). After the removal of 385 duplicates, 586 records were left for title and abstract screening. 105 full-text articles were accessed with 10 articles meeting the eligibility criteria (Fig. 1).

To place African research in a global context, we ran an exploratory PubMed search using the MeSH terms [‘breast neoplasm AND ‘genetics’]. This broad query retrieved 90,735 publications. Re-running the same query but appending the names of all 54 African countries reduced the yield to 1631 records that potentially involved African data.

### Characteristics of included studies

**Fig. 2:**
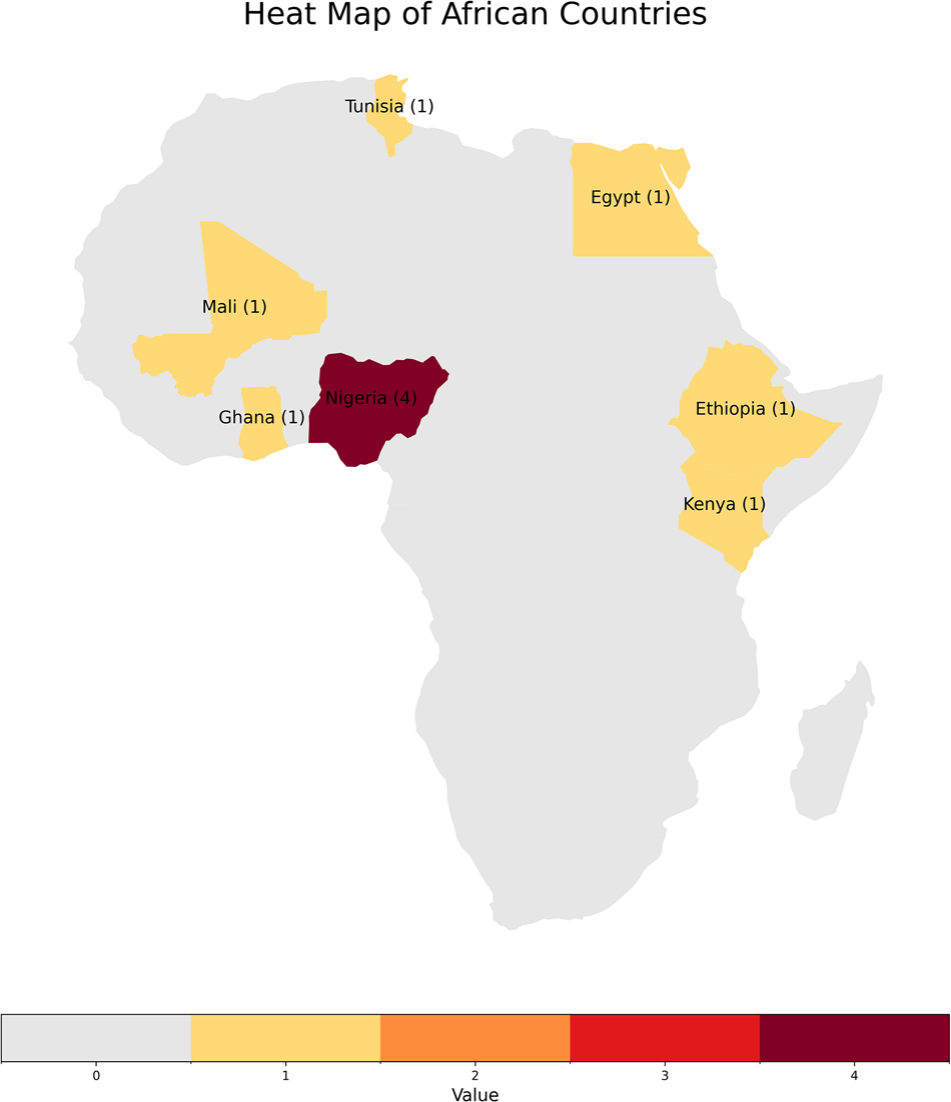
Publications included in the systematic review by country.

The 10 somatic-focused studies identified were conducted in seven African countries: four from Nigeria^12–15^, and each one from Kenya^16^, Tunisia^17^, Ethiopia^18^, Egypt^19^, Ghana^20^, and Mali^21^ (Fig. 2 and Table 1).

**Table 1:** Characteristics of studies

Author*/year	Country	Cohort size	Mean age (SD)	Sequencing platform	Sample source	Comparison cohort
Tang^16^ 2023	Kenya	23	48.8	WES	Frozen biopsy cores	Mixed USA cohort
Barbirou^17^ 2022	Tunisia	32	42.94 (4.25)	WGS	Cell-free DNA	None
Hercules^12^ 2022	Nigeria	31	49.9	WES	FFPE	TCGA
Ansari-Pour^13^ 2021	Nigeria	97	50.8(12.8)	WGS, RNA- Seq	Blood + biopsy cores	TCGA
Schwartz^18^ 2021	Ethiopia	3	Unreported	WES	FFPE	TCGA
Kassem^19^ 2021	Egypt	8	40 (7.01)	Cancer hotspot panel	Liquid biopsy (ctDNA)	None
Anwar^20^ 2020	Ghana	11	48	Comprehensive Cancer Panel (Ion Ampliseq CCP)	FFPE	None
Wang 2019^14^	Nigeria	170	51.3(12.7)	WES, WGS	Blood + biopsy cores	TCGA
Pitt^15^ 2018	Nigeria	194	51.3(12.7)	WGS, WES, RNA-Seq	Blood + biopsy cores	TCGA
Ly^21^ 2013	Mali	28	49	CGH array	FFPE	USA

*Only the first author is named for each study.

Six of the studies characterised somatic driver mutations, an equal number included an analysis on copy number variations (CNVs) and 5 studies described mutational signatures. Ly et al.^21^ in Mali and Anwar et al.^20^ in Egypt focused entirely on profiling CNVs. Six studies used either circulating tumour DNA (ctDNA)^17,19^ or biopsy cores^13–15,22^, while the rest used formalin-fixed paraffin-embedded (FFPE) tissue as their sample source. Studies that incorporated comparative cohorts utilised TCGA that provided both European and African/African American sample data with two studies choosing cohorts from medical centres in the United States^16,21^. The mean ages of the participants clustered within a narrow range of late 40 s to early 50 s.

There was a substantial enrichment for ER-/PR-/HER2- tumours, that is, TNBCs, comprising between 41% and 60% of all samples across all studies that provided the data. In contrast, Ethiopian, Egyptian and Kenyan patients presented a higher presence of ER+ positive tumours.

### Risk of bias

The risk of bias for the included cohort studies was evaluated using the nine-point Newcastle-Ottawa Scale (NOS), which assesses three domains: selection of participants, comparability of cohorts, and assessment of outcomes. Five studies (Barbirou et al., Hercules et al., Ansari-Pour et al., Wang et al., and Pitt et al.)^12–15,17^ achieved a total score of 7 or above and were rated as ‘High’ quality, indicating a low risk of bias. Five studies were rated as ‘Moderate’ quality (Tang et al., Schwartz et al., Kassem et al., Anwar et al. and Ly et al.)^16,18–21^ with scores between 4 and 6 indicating moderate risk of bias, while no study was rated as low quality. Common strengths included multi-centre cohorts, high-quality pathology and sequencing pipelines. The most frequent limitations contributing to moderate risk were lack of adjustment for confounding variables such as tumour subtypes and inadequate reporting on genomic sample attrition—loss of eligible samples due to insufficient tumour content or failed quality control. These methodological concerns could potentially introduce bias in the estimation of exposure-outcome relationships. Table 2 summarises the NOS quality ratings and component scores for each included study.

**Table 2:** Quality assessment of included cohort studies

Newcastle-Ottawa Scale Cohort Studies									
Study	Selection				Comparability	Outcome		Total	Quality Rating
	Representativeness of cohort	Selection of controls	Ascertainment of exposure	Baseline outcome of interest	Control on key confounders	Assessment of Outcome	Adequacy of follow-up on cohorts		
Tang et al.^16^	*	*	*	*		**		6	Moderate
Barbirou et al.^17^	*	*	*	*		**	*	7	High
Hercules et al.^12^	*	*	*	*		**	*	7	High
Ansari-Pour et al.^13^	*	*	*	*		**	*	7	High
Schwartz et al.^18^	*	*	*	*		**		6	Moderate
Kassem et al.^19^			*	*		*	*	4	Moderate
Anwar et al.^20^	*		*	*		*		4	Moderate
Wang et al.^14^	*	*	*	*	**	**	*	9	High
Pitt et al.^15^	*	*	*	*	*	**	*	8	High
Ly et al.^21^	*	*	*	*		**		6	Moderate

Each “*” indicates one point awarded according to the Newcastle-Ottawa Scale criteria.

### Somatic driver mutations

**Fig. 3:**
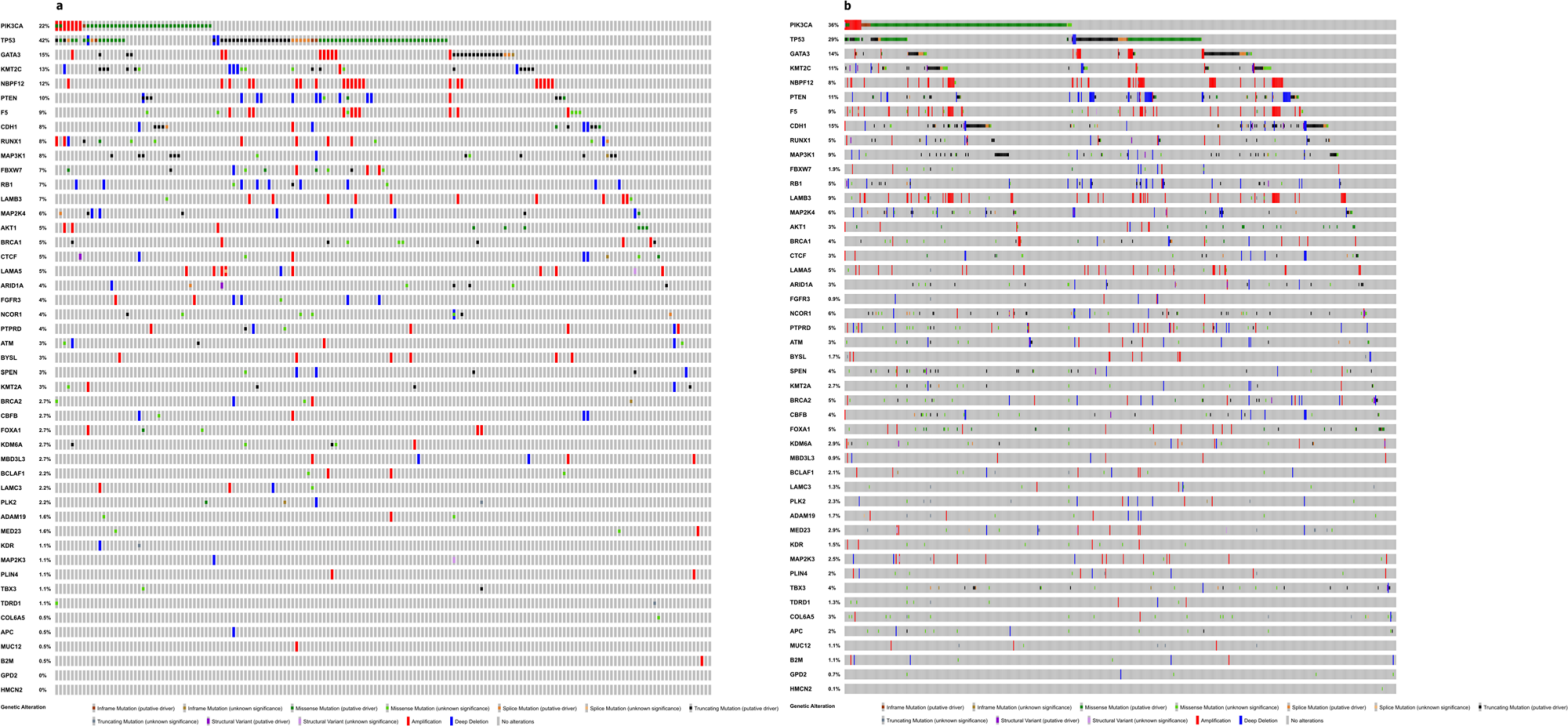
Landscape of somatic driver genes in breast cancer identified in the selected African studies showing their representation against TCGA pancancer atlas. **a** TCGA Black/African American participants (*n* = 178) and (**b**) TCGA White participants (*n* = 739). Matrix of coloured tiles depict each TCGA sample’s alteration type (see key).

**Fig. 4:**
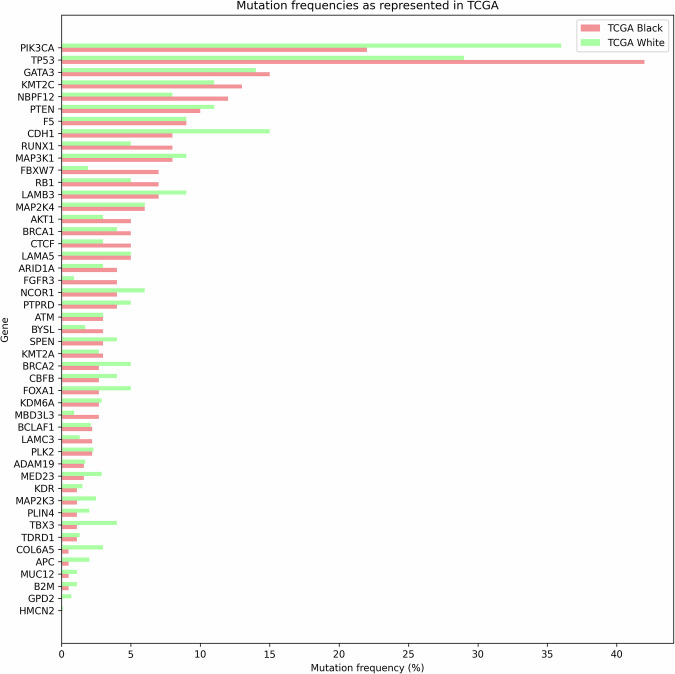
Breast cancer somatic driver genes mutation frequencies (*n* = 47) identified in the reviewed African studies showing their representation against TCGA subgroups. Green bar is the percentage mutated for TCGA White and orange bar for TCGA Black. Capturing similarities and differences across the two groups.

Six studies reported driver mutations from Nigerian (3)^12–14^, Ethiopian^18^, Egyptian^19^ and Kenyan^16^ cohorts. These six studies reported a total of 47 mutations occurring above a 2% frequency threshold. To contextualise these findings, we compared these mutation frequencies against the entire breast invasive carcinoma dataset available on the TCGA PanCancer Atlas^23^. Figure 3 displays an oncoprint of the 47 driver genes showing the alteration types and frequencies in the TCGA Black and TCGA White cohorts while Fig. 4 presents side by side bar charts of those mutation frequencies for direct comparison.

**Fig. 5:**
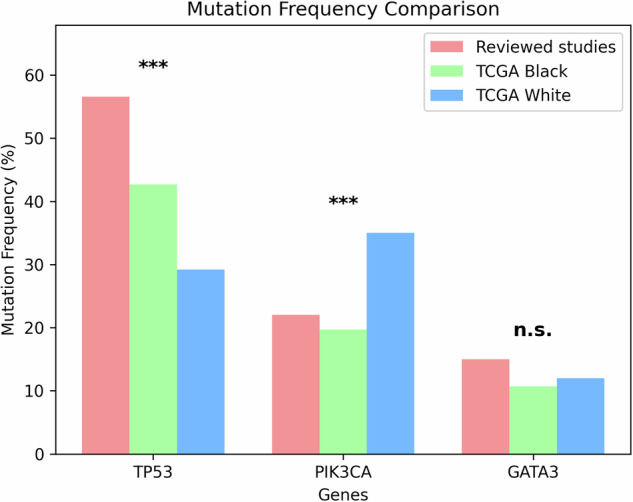
**Breast cancer somatic driver genes that occurred at a mean mutation frequency of >10% in the participants across all reviewed studies with available data vs TCGA pancancer atlas Black and White patients**. Chi-square test significance levels: *p* < 0.05 (*),(*), *p* < 0.01 (**), *p* < 0.001 (***), not significant(n.s.). Bonferroni correction (α = 0.0167).

**Fig. 6:**
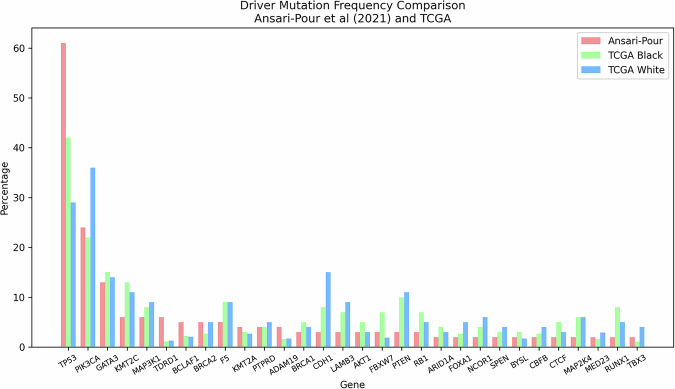
Somatic driver mutation frequencies in the Ansari-Pour et al. (2021) Nigerian cohort versus TCGA reference groups. Bar heights show the percentage of tumours harbouring mutations in each gene for the Ansari-Pour series (red), TCGA Black/African American (green) and TCGA White (blue) participants. Genes are ordered by decreasing mutation rate in the Ansari-Pour cohort. Two-sample proportion z-tests (Ansari vs. TCGA White) revealed highly significant enrichment of *TP53* and *TDRD1*, and depletion of *CDH1* (all *p* < 0.001 after Bonferroni correction). *PIK3CA* was also less frequent in the Ansari cohort at an unadjusted threshold (*p* = 0.0168). Other genes showed no statistically significant differences.

We focused on the handful of canonical breast-cancer drivers that were mutated in more than 10% of tumours across the African cohorts—namely *TP53*, *PIK3CA* and *GATA3*—and directly benchmarked their prevalence against the TCGA White dataset. On average, *TP53* mutations occur in 57% ( ± 15%) of African cases versus 29% of TCGA White tumours—a 28% increase, *p* < 0.001—while *GATA3* mutations average 15% ( ± 2.8%) compared with 12% in the White cohort—a 3% increase, *p* = n.s.). Conversely, *PIK3CA* mutations are less common in African cohorts, averaging 22% ( ± 3.6%) versus 35% in TCGA White ( − 13%, *p* < 0.001) (Fig. 5). These consistent shifts underscore both the elevated prevalence of *TP53* and *GATA3* alterations and the relative paucity of *PIK3CA* mutations in African breast cancers when benchmarked against a predominantly European-ancestry reference Fig. 6.

Nigerian breast-cancer cohorts exhibit a markedly divergent somatic landscape compared with the TCGA White reference: *TP53* is mutated in 58% of Nigerian tumours versus 29% in TCGA White^12,13,15^, while *PIK3CA* alterations—though similar to those reported in Kenyan and TCGA Black cohorts—remain substantially lower than in TCGA White^16^. Loss-of-function events in *CDH1* and *PTEN* are also underrepresented in Nigerian cases^13^. Two landmark studies have since expanded the African driver-gene repertoire: Pitt et al.^15^ identified four novel significantly mutated genes—*PLK2*, *B2M*, *KDM6A* and *GPS2*—with *PLK2* and *KDM6A* alterations particularly enriched in HER2^+^ tumours, and Ansari-Pour et al.^13^ uncovered five additional candidates—*ADAM19*, *LAMB3*, *BYSL*, *F5* and *TDRD15*—with *BYSL*, *F5* and *TDRD15* displaying tumour-suppressor–like mutational patterns. In parallel, Kenyan cohorts demonstrated an enrichment of *ARID1A* mutations (17%), underscoring both shared and population-specific somatic events across sub-Saharan breast cancers^16^.

In Ansari-Pour et al.’s Nigerian cohorts (Fig. 6), within-cohort analyses revealed *TP53* enrichment in ER– tumours, sustained *GATA3* enrichment after adjusting for receptor status, and higher HRD-signature activity in TNBC versus other subtypes—yet no cross-cohort, subtype-matched comparison was performed. In contrast, re-analysis of TCGA shows that Black patients harbour more *TP53* mutations (46% vs. 29%) and fewer *PIK3CA* mutations (20% vs. 36%) than White patients—while *GATA3* rates are virtually identical (∼10% each)—but that all three differences disappear when cases are stratified by IHC subtype, demonstrating they reflect subtype distribution rather than ancestry^15^. By comparison, Nigerian HR + /HER2 – cohort, *TP53* (62% vs. 46%/29%), *GATA3* (17% vs. 10%/9.5%) and *PIK3CA* (17% vs. 20%/36%) mutation rates remain significantly different from both TCGA Black and White groups even within the same subtype, indicating an ancestry-linked somatic profile beyond subtype effects.

### Copy number alterations (CNAs)

CNV data was available for Nigerian (3 studies)^12,13,15^, Ghanian^20^, Malian^21^ and Tunisian patients^17^ (1 study each). Anwar et al^20^ identified 17 genes in 90% of the Ghanian tumours that had recurrent CNV’s with the most frequent gains observed in *RECQL4* (8q24.3) and *SDHC* (1q23.3) genes in 50% and 60% of cases, respectively. The study also noted an overexpression of EZH2 a key epigenetic regulator that represses tumour suppressor genes and promotes cancer progression, particularly in aggressive subtypes such as triple-negative breast cancer^26^.

Compared to the American cohort used in the study, Malian women exhibited significantly more frequent gains in chromosomal regions, namely 6p21,9q34, 11q13, 12q24, 17q25 and 22q12.1-22q13.1. These regions contained several drug-targetable genes including *EWSR1*, *BIRC5*, *BCR*, *VAV2* and *ABL1*. 53% of the patients had an amplification of the *BCR* gene region. Although best known for its fusion with *ABL1* in leukaemia, recent studies suggest that *BCR* overexpression may also promote breast tumour progression through modulation of MAPK signalling pathways. Notably, 76% of the TNBC patients in the study exhibited elevated BCR protein expression^21^.

In a Tunisian cohort, authors utilised a circulating free DNA (cfDNA) assay to evaluate its utility as a non-invasive early diagnostic approach in individuals with dense breast tissue^17^. Rather than identifying novel alterations, the study prioritised the comparison of CNA’s in established breast cancer-associated genes as well as small variants in a panel of genes^26^. Notably amplified regions included the *KSR2*, *CANT1*, *MSI2* and *MAP2K4* genes, all of which have been implicated in tumour progression, cell signalling, or regulation of proliferation. Additionally, the study examined single nucleotide polymorphisms (SNPs) with putative oncogenic relevance, although these were not functionally validated.

**Fig. 7:**
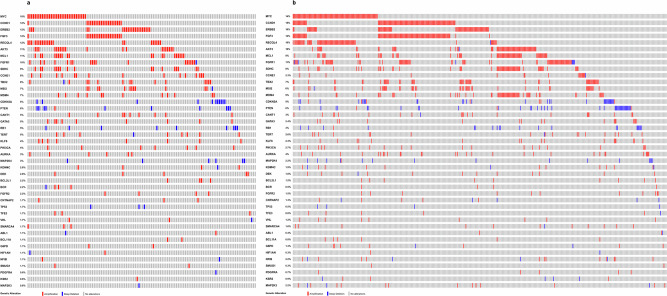
Copy number alterations in reviewed studies shown against their representation in the TCGA pancancer atlas. **a** TCGA Black patients (*n* = 181) and (**b**) TCGA White patients (*n* = 741). Coloured matrix details amplifications and deletions (see key).

Nigerian breast cancer genomes show a markedly higher structural variant (SV) burden—averaging ~551 SVs in HR + /HER2− and ~626 in triple-negative tumours—than both TCGA Black and White cohorts^15^. These tumours also display elevated homologous recombination deficiency (HRD) signatures and a higher prevalence of TP53 mutations, particularly in HR + /HER2− subtypes^15^. Consistently, there were greater proportions of whole genome duplication and genomic instability (3-fold)^13^. In the same patients, notable alterations included deletions in regions overlapping *TP53* and amplification of *ERBB2* genes in 63% and 24% of the samples respectively^15^. HR-/HER2+ tumours characteristically had a loss of heterozygosity (LOH)at 14q in 58% of the patients compared to 7% in TCGA White^13^. Figure 7 illustrates the genomic regions that were identifiable in the studies and the comparison TCGA data (Fig. 7).

### Mutational signatures

Somatic mutational signatures, which are recurring patterns of somatic mutations reflecting distinct endogenous or exogenous mutagenic processes^27^, were described in 4 Nigerian studies^12–15^ and 1 Kenyan study^16^. Among Kenyan patients, Catalogue of Somatic Mutations in Cancer (COSMIC) single base substitution signatures (SBS)1, SBS2, SBS3, SBS5, SBS13, and SBS18 were identified^16^.

In contrast Nigerian patients exhibited a broader range of enriched signatures. Among the mutational signatures identified were SBS1, SBS2, SBS3, SBS8–10, SBS13, SBS17, SBS18, SBS24, SBS39, and indel signatures ID6 and ID8, reflecting diverse mutational processes. SBS1 marks age-related changes, SBS2/13 reflect APOBEC activity, and SBS3/ID6 are indicative of homologous recombination deficiency (HRD). SBS10 relates to *POLE* mutations, while SBS17/18 suggest oxidative or alkylating damage. SBS8/39 are of unclear origin but recurrent, and ID8 is linked to mismatch repair deficiency. SBS24, rarely seen, may signal environmental exposure (e.g., aflatoxin)^12–15^. Mutational signatures in Nigerian tumours also showed a shift toward higher HRD activity and lower APOBEC mutagenesis, especially in the HR + /HER2− subtype. This pattern was linked to driver mutations: *TP53* and *BRCA1/2* correlated with HRD, while *PIK3CA* and *CDH1* correlated with APOBEC activity. Notably, elevated HRD in Nigerians persisted after adjusting for age and these mutations, suggesting additional ancestry-related genomic factors^13^. Of note, SBS3, was less prominent in Kenyan samples compared to African American (AA) cohorts, suggesting lower HRD activity in the Kenyan group^16^.

Mutational signatures underscore the genomic diversity within African populations and may help stratify patients by therapeutic vulnerabilities. For instance, Nigerian patients may benefit from HRD-targeted therapies, such as PARP inhibitors, which exploit deficiencies in DNA repair mechanisms or APOBEC-driven immunogenicity relevant to checkpoint blockade therapies^13^.

The provisional identification of ancestry-associated mutational signatures highlights the potential value of more inclusive cancer genomics research, which could inform the development of population-adapted therapeutic strategies.

## Discussion

Of 90,735 breast carcinoma genetics papers (2004–2024), fewer than 2% (*n* = 1631) focused on African populations, and only 7( ≈ 10%) of 54 African nations have reported tumour-sequencing studies. This substantial research gap underscores a systemic underrepresentation of African genomic data, limiting the generalisability of global breast cancer findings and the development of ancestry-informed diagnostics and therapeutics.

To address this gap, several initiatives have been launched. The Human Heredity and Health in Africa (H3Africa) Consortium, includes cancer-related projects such as the African Female Breast Cancer Epidemiology (AFBRECANE) study^28^, which enrolled over 1000 Nigerian breast cancer patients to investigate genetic and environmental risk factors. Additionally, the African Cancer Genome Registry (ACGR)^29^ focuses on breast and prostate cancers across African and Caribbean populations, integrating whole exome sequencing, gene panel testing, and clinical annotation to identify ancestry-specific genetic drivers. These initiatives aim to expand research capacity and generate population-specific data to support clinical translation and reduce global disparities in cancer outcomes.

TNBC disproportionately affects Black women and presents at a younger age vs. European cohorts. Its five-year mortality risk is 25%, rising to 50% for stage III diagnoses^30^. However, the proportion of TNBC varies markedly across African countries, ranging from ~15% in Central Africa to over 40% in West Africa, a variability partly driven by differences in immunohistochemistry (IHC) protocols and thresholds for ER/PR/HER2 positivity^31^. These between-country disparities in TNBC may themselves account for some of the observed differences in somatic mutation frequencies. Additionally, germline pathogenic variants, particularly in *BRCA1* and *BRCA2*, are more prevalent in certain African populations. For instance, there is a 7–11% *BRCA1/2* mutation carrier rate reported in Nigerian and South African cohorts^24,25^, which further elevates TNBC risk and may bias somatic mutations toward HRD subtypes^32,33^.

Mutational profiling of African breast tumours revealed higher frequencies of alterations in *TP53* and *GATA3*. These regulators of cell proliferation also define intrinsic breast cancer subtypes, with direct implications for prognosis and treatment response^34^. Notably, *TP53* mutations—closely linked to genomic instability, elevated tumour mutational burden (TMB) and aggressive phenotypes—were significantly more common in African than in European cohorts. Such ancestry-associated mutational patterns likely contribute to the observed disparities in clinical outcomes and therapeutic efficacy.

Conversely, *PIK3CA* mutations, which are commonly observed in HR+ tumours and serve as therapeutic targets in many settings, were less frequent Although less prevalent than TNBC, HR^+^/HER2^−^ tumours in African cohorts exhibited a high burden of structural variants alongside pronounced homologous-recombination-deficiency (HRD) signatures. These features suggest potential sensitivity to PARP inhibitors and hormone‑directed therapies^35^, warranting further subtype‑specific investigation. These findings reinforce the clinical value of somatic mutation profiling, particularly in resource-limited settings where comprehensive molecular diagnostics remain underutilised. Analogous to the use of *BRAF* mutations in melanoma and *EGFR* in lung cancer, *TP53* and *PIK3CA* status could inform eligibility for precision therapies or inclusion in clinical trials^36^. Integrating such molecular markers into standard-of-care protocols in Africa will require both technological investment and capacity-building initiatives.

High tumour mutational burden (TMB) has emerged as a key biomarker for immunotherapy, since a greater number of somatic mutations increases the likelihood of neoantigen presentation and T-cell recognition. In all African cohorts that measured TMB, levels were consistently elevated compared with non-African groups, suggesting enhanced tumour immunogenicity and potential suitability for immune checkpoint inhibitors—particularly in TNBC^37^. Although high TMB does not uniformly predict response, its persistent elevation in African-derived samples underscores the promise of immunotherapeutic strategies and the need for prospective validation.

CNA analyses revealed a general trend toward more frequent gains than losses in African-derived tumours^17,20,21^. These gains frequently involve genes central to oncogenic signalling, transcriptional control, and cell cycle progression. For example, recurrent amplification of *ERBB2*, may contribute to tumour aggressiveness and poor prognosis^35^. The identification of novel, potentially ancestry associated, CNA regions warrant deeper mechanistic exploration and could offer novel therapeutic targets if functionally validated^17,21^.

A major challenge in interpreting the elevated *TP53/GATA3* and reduced *PIK3CA* rates in African-ancestry cohorts is disentangling true ancestry effects from differences in subtype mix. Ansari-Pour et al. showed within-cohort subtype associations—*TP53* enrichment in ER-tumours and persistent *GATA3* enrichment after adjusting for receptor status—but did not perform cross-cohort, subtype-matched comparisons^13^. Indeed, in TCGA the higher *TP53* and lower *PIK3CA* rates in Black versus White patients vanish once stratified by IHC subtype, confirming subtype distribution as the driver of those disparities^15^. By contrast, Nigerian HR + /HER2 – tumours maintain significantly higher *TP53* and *GATA3* and lower *PIK3CA* frequencies even within the same subtype, suggesting a genuine, ancestry-linked mutational profile^15^. Going forward, uniformly annotated receptor status and direct, subtype-matched, cross-cohort analyses will be essential to isolate tumour-intrinsic ancestry effects.

Somatic mutational signatures provide insight into the underlying biological processes driving tumour development and may reflect both intrinsic factors, such as inherited DNA repair defects, and extrinsic exposures, including environmental mutagens^38^. One promising application of this information is the use of homologous recombination deficiency (HRD) as a stratification tool for therapy, particularly in triple-negative breast cancer (TNBC). This approach is supported by findings in Nigerian cohorts, where elevated levels of HRD-associated signatures were observed, suggesting that a subset of patients may benefit from therapies targeting defective DNA repair mechanisms, such as PARP inhibitors. As sequencing data from African populations continues to grow, it is likely that novel previously uncharacterised signatures and exposures will emerge, further refining our understanding of tumour biology and informing tailored treatment strategies.

While the rich ancestral diversity of African cohorts is a strength, it also introduces variability in somatic mutation profiles and structural variants that can complicate cross-study comparisons, as evidenced by the findings in these studies. The majority of studies included in this review analysed Nigerian cohorts, whose mutational profiles demonstrate significant overlap with African American (AA) populations, likely attributable to shared West African ancestry^39^. Shared West‑African ancestry between Nigerian and African‑American cohorts explains some overlap in mutation patterns, but underrepresented North, East, and Southern African populations may harbour distinct alterations. For instance, preliminary findings from Uganda have identified a higher rate of *BRCA1/2* Mutations, which may guide the use of PARP inhibitors^32^.

Our review has several important limitations. First, we identified only 10 somatic-focused sequencing studies spanning just seven of 54 African countries, with nearly half of them from Nigeria, which constrains the geographic and ethnic representativeness of our findings. Second, cohort sizes varied widely—and in some cases were very small—limiting statistical power and the ability to generalize mutation frequencies to national populations. Third, the included studies used a wide array of sequencing approaches—from WGS/WES to medium- and small-targeted panels (including cfDNA assays)—each with distinct gene content, coverage depth and tissue input (FFPE vs. fresh), and they applied diverse bioinformatic pipelines (alignment, variant callers, filtering thresholds) and driver-calling frameworks (candidate-gene lists, MutSigCV, dNdScv, etc). This methodological heterogeneity, coupled with inconsistent reporting of sample quality metrics and confounder adjustment, has severely impeded direct cross-study comparisons.

Finally, the absence of a formal meta-analysis and the variable depth of genomic coverage across studies prevented quantitative synthesis of mutation prevalences or effect sizes. Additionally, both inter- and intra-tumour heterogeneity^40^ appear to be more pronounced in African breast cancer cases relative to European counterparts, further complicating the identification of universally applicable biomarkers. Together, these factors underscore the need for larger, multi-centre, harmonized sequencing efforts across diverse African populations to ensure consistency, reproducibility, and equitable integration into precision oncology.

The observed mutational patterns and somatic alterations among African breast cancer patients suggest meaningful biological differences with potential clinical relevance. Continued investment in large-scale, prospective cohorts with well-matched controls will be essential to validate emerging insights and facilitate the development of risk stratification tools, diagnostic assays, and therapeutics tailored to African populations.

## Methods

This systematic review was conducted per the guidelines outlined in the Preferred Reporting Items for Systematic Reviews and Meta-Analyses (PRISMA) statement^41^. The protocol was registered in PROSPERO (CRD42024549600).

### Study eligibility

The eligibility criteria were based on the disease of interest, patient population and the type of analysis conducted. We included studies that investigated primary breast cancer in female African patients and used next-generation sequencing methods to characterise somatic mutations in tumour tissue or cell free tumour DNA.

Studies focusing on germline mutations were excluded to maintain a clear focus on tumour-acquired mutations. Due to its low incidence and distinct molecular profile, male breast cancer was not included in the analysis. Additionally, research with non-genomic endpoints such as public health knowledge, attitudes and practices, clinical care, medical risk reduction techniques, immunology-based assays, reviews, case studies, and correspondence were excluded as they did not directly contribute to the primary objective of examining the somatic genome of female breast cancer in African populations.

### Search approach

To retrieve all relevant studies, four databases were chosen due to their comprehensive coverage of biomedical literature, interdisciplinary scope, and rigorous indexing standards. The peer-reviewed publications incorporated in this review were obtained from PubMed, Scopus, Embase and Web of Science (WOS).

The database search covered the period of 1 January 2004 to 30 September 2024. The searches were limited to publications from 2004 corresponding to the introduction of next-generation sequencing (NGS) platforms enabling whole genome and whole exome sequencing^42^. In all four databases, comprehensive search strategies that combined controlled terminology and synonyms for “breast carcinoma”, “genetics” and “mutation” and explicitly listed every African country by name. PubMed Medical Subject Headings (MeSH) terms were utilised as they provide more accurate and relevant search results while overcoming synonymy and polysemy^43^ (Supplementary S1). For Scopus, Web of Science and Embase, their built-in search functions were used to obtain the records. The search terms and strategies incorporated for the three databases are included in Supplementary S2–S4. The search results were then exported following custom record fields selections for article title, authors, publication year and abstract.

Biopython^44^, an open-source tool specifically designed to interact with the PubMed via the National Centre for Biotechnology Information (NCBI) Entrez system was used to retrieve abstracts and metadata. This tool was used because a direct PubMed export yielded poorly formatted abstracts. A search query was created using ‘Entrez.esearch‘ function, which retrieved PubMed article IDs based on the MeSH search terms and publication date limits previously detailed. The XML data obtained was then parsed to similarly obtain the article titles, authors, publication year and abstract. The PubMed records identified were then combined into a single corpus with those from Scopus and WOS for duplicate removal and screening.

### Data screening and extraction

Natural language processing (NLP) techniques were used for text mining to extract the country of study and the type of genomic analysis conducted from the abstracts of the articles. NLP was particularly beneficial in streamlining the extraction process by quickly identifying relevant details, reducing manual screening workload, and enhancing accuracy. The ‘spaCy’ python module^45^ was used as it contains a library with named entity recognition (NER) capabilities for identification of the countries mentioned in the titles and abstracts.

NER and genomic analyses keywords specific to this search; ‘sequencing’, ‘RNAseq’, ‘RNA sequencing’,‘germline’, ‘somatic’, ‘brca’ and ‘genome wide association’, were used to create pattern tokens to map articles to their respective countries and either ‘germline’ or ‘somatic’. This was further validated manually. A data extraction sheet was used to collate information on first authors, publication year, study location, breast cancer subtypes, sequencing methodology and the genetic variants reported from the full texts of the included studies.

### Risk of bias assessment

The quality of included studies was assessed using a modified Newcastle-Ottawa Scale (NOS)^46,47^, covering three main domains: selection, comparability and outcome. The NOS scale assigned a maximum of nine points for various components including cohort representativeness, quality-controlled specimens, adjustment for tumour subtype and rigour of the genomic assay. Studies scoring seven or more points (≥7) were deemed high quality, those scoring four to six points (4-6) were classified as moderate quality, and those scoring fewer than four points (<4) were considered low quality.

### Data synthesis

We conducted a structured narrative synthesis, in line with established methodological guidance^48^. All extracted African female breast cancer mutational data outcomes—including somatic driver mutations, copy number variations, and mutational signatures—were tabulated by country and immunohistochemical subtype (ER/PR/HER2). These data were descriptively compared to the corresponding proportions in the TCGA Pan-Cancer Atlas^23^ racial categories “Black or African American” and “White,” which broadly represent individuals of African (including African American) and European descent, respectively. We acknowledge that these broad racial and ancestral categories—adopted from TCGA—do not fully capture the complexity of genetic ancestry or sociocultural identity; hence, comparisons must be interpreted with caution^49^.

The TCGA dataset was selected for comparison as it is a publicly available pan-cancer resource offering integrated multi-omic and clinical data across ancestrally diverse populations. Data were accessed through the cBioPortal for Cancer Genomics (available at https://www.cbioportal.org/)^50^. For clarity purposes, TCGA participants of African descent or African American will be termed as ‘TCGA Black’ and participants of European ancestry or European American as ‘TCGA White’.

Where possible, chi-square testing (χ²) was used to determine statistical significance of the mutation rates between the reviewed studies and TCGA cohorts. Mean mutation rates ( ± SD) are reported for the included studies. P-values were adjusted for multiple comparisons using the Bonferroni correction^51^. All statistical analyses were carried out in Python. Due to heterogeneity in study designs, NGS panels, sequencing coverage, and bioinformatic pipelines, formal meta-analysis or statistical pooling was not possible. Instead, we highlighted recurrent mutations reported across multiple studies and contrasted their frequencies with TCGA reference cohorts, discussing concordant and discordant patterns in a narrative format.

## Supplementary information

Supplementary Information

## Data Availability

Template data extraction forms, the datasets used for analysis, and the underlying code are not publicly accessible but can be obtained from the corresponding author upon reasonable request.
