# Supplementary material for: Genomic landscapes of breast cancer in African populations: a systematic review
Source: NPJ Breast Cancer. 2025 Aug 14;11:91. doi: 10.1038/s41523-025-00809-9 (PMC12354838; doi:10.1038/s41523-025-00809-9)
Supplement: Supplementary file 1 — Supplementary Information [file 41523_2025_809_MOESM1_ESM.pdf]

Supplementary Materials

Table S1: PubMed search strategy using MeSH descriptors

1	[“Breast neoplasms”] OR Breast Carcinoma In Situ OR Breast Neoplasms, Male OR Carcinoma, Ductal, Breast OR Carcinoma, Lobular OR Hereditary Breast and Ovarian Cancer Syndrome OR Inflammatory Breast Neoplasms OR Triple Negative Breast Neoplasms OR Unilateral Breast Neoplasms<br><br>AND
2	(Africa OR Algeria OR Angola OR Benin OR Botswana OR "Burkina Faso" OR Burundi OR "Cape Verde" OR Cameroon OR "Central African Republic" OR Chad OR Comoros OR "Congo (Democratic Republic)" OR "Congo (Republic)" OR Djibouti OR Egypt OR "Equatorial Guinea" OR Eritrea OR Eswatini OR Ethiopia OR Gabon OR Gambia OR Ghana OR Guinea OR "Guinea-Bissau" OR "Ivory Coast" OR Kenya OR Lesotho OR Liberia OR Libya OR Madagascar OR Malawi OR Mali OR Mauritania OR Mauritius OR Morocco OR Mozambique OR Namibia OR Niger OR Nigeria OR Rwanda OR "São Tomé and Príncipe" OR Senegal OR Seychelles OR "Sierra Leone" OR Somalia OR "South Africa" OR "South Sudan" OR Sudan OR Tanzania OR Togo OR Tunisia OR Uganda OR Zambia OR Zimbabwe)<br>AND
3	[Genetics] OR Cytogenetics OR Genetic Research OR Genetics, Behavioural OR Genetics, Microbial OR Genetics, Population OR Genomics OR Human Genetics OR Immunogenetics OR Molecular Biology OR Pharmacogenetics OR Phenomics OR Radiation Genetics OR Toxicogenetics<br><br>AND
4	[Mutation] OR Allelic Imbalance OR Base Pair Mismatch OR Chromosome Aberrations OR Codon, Nonsense OR DNA Repeat Expansion OR Frameshift Mutation OR Gain of Function Mutation OR Gene Amplification OR Gene Duplication OR Genomic Instability OR Germ-Line Mutation OR INDEL Mutation OR Loss of Function Mutation OR Mutagenesis, Insertional OR Mutation Accumulation OR Mutation Rate OR Mutation,

	Missense OR Point Mutation OR Sequence Deletion OR Sequence Inversion OR Silent Mutation OR Suppression, Genetic OR Synthetic Lethal Mutations

Table S2: Web of science search strategy

1	(((ALL=(("breast cancer" OR "breast carcinoma" OR "breast neoplasm*"))) AND AB=(Africa OR Algeria OR Angola OR Benin OR Botswana OR "Burkina Faso" OR Burundi OR "Cape Verde" OR Cameroon OR "Central African Republic" OR Chad OR Comoros OR "Congo (Democratic Republic)" OR "Congo (Republic)" OR Djibouti OR Egypt OR "Equatorial Guinea" OR Eritrea OR Eswatini OR Ethiopia OR Gabon OR Gambia OR Ghana OR Guinea OR "Guinea-Bissau" OR "Ivory Coast" OR Kenya OR Lesotho OR Liberia OR Libya OR Madagascar OR Malawi OR Mali OR Mauritania OR Mauritius OR Morocco OR Mozambique OR Namibia OR Niger OR Nigeria OR Rwanda OR "São Tomé and Príncipe" OR Senegal OR Seychelles OR "Sierra Leone" OR Somalia OR "South Africa" OR "South Sudan" OR Sudan OR Tanzania OR Togo OR Tunisia OR Uganda OR Zambia OR Zimbabwe)) AND ALL=(genetic* OR genomic*)) AND ALL=(mutation*)

Table S3: Scopus search strategy

1	TITLE-ABS-KEY(("breast cancer" OR "breast neoplasm" OR "breast carcinoma") AND (Africa OR Algeria OR Angola OR Benin OR Botswana OR "Burkina Faso" OR Burundi OR "Cape Verde" OR Cameroon OR "Central African Republic" OR Chad OR Comoros OR "Congo (Democratic Republic)" OR "Congo (Republic)" OR Djibouti OR Egypt OR "Equatorial Guinea" OR Eritrea OR Eswatini OR Ethiopia OR Gabon OR Gambia OR Ghana OR Guinea OR "Guinea-Bissau" OR "Ivory Coast" OR Kenya OR Lesotho OR Liberia OR Libya OR Madagascar OR Malawi OR Mali OR Mauritania OR Mauritius OR Morocco OR Mozambique OR Namibia OR Niger OR Nigeria OR Rwanda OR "São Tomé and Príncipe" OR Senegal OR Seychelles OR "Sierra Leone" OR Somalia OR "South Africa" OR "South Sudan" OR Sudan OR Tanzania OR Togo OR Tunisia OR Uganda OR Zambia OR Zimbabwe) AND (genetic* OR genomic*) AND (mutation*)) AND PUBYEAR > 2003 AND PUBYEAR < 2025

Table S4: Embase search strategy

1	(('breast cancer' or 'breast neoplasm' or 'breast carcinoma') and ((('africa' or 'algeria' or 'angola' or 'benin' or 'botswana' or 'burkina faso' or 'burundi' or 'cape verde' or 'cameroon' or 'central african republic' or 'chad' or 'comoros' or 'congo democratic republic' or 'congo republic' or 'djibouti' or 'egypt' or 'equatorial guinea' or 'eritrea' or 'eswatini' or 'ethiopia' or 'gabon' or 'gambia' or 'ghana' or 'guinea' or 'guinea-bissau' or 'ivory coast' or 'kenya' or 'lesotho' or 'liberia' or 'libya' or 'madagascar' or 'malawi' or 'mali' or 'mauritania' or 'mauritius' or 'morocco' or 'mozambique' or 'namibia' or 'niger' or 'nigeria' or 'rwanda' or 'sao tome) and principe') or 'senegal' or 'seychelles' or 'sierra leone' or 'somalia' or 'south africa' or 'south sudan' or 'sudan' or 'tanzania' or 'togo' or 'tunisia' or 'uganda' or 'zambia' or 'zimbabwe') and (genetic* or genomic*) and mutation*).af.
	limit 1 to yr="2004 - 2024"

Table S5: somatic driver genes (n=47) identified per country at a cutoff of >2% frequency. For studies that incorporated analysis of mutational drivers . (x) marks named driver genes in the studies based on different countries.

Gene	Country (number of studies)			
	Nigeria (n=3)	Kenya (n = 1)	Ethiopia (n=1)	Egypt (n =1)
TP53	x	x		x
PIK3CA	x	x		x
GATA3	x			
KMT2C	x			
MAP3K1	x			
TDRD15	x			
BCLAF1	x			
BRCA2	x		x	
F5	x			
KMT2A	x			
PTPRD	x			
ADAM19	x			
BRCA1	x			
CDH1	x			
LAMB3	x			
AKT1	x			
FBXW7	x			
PTEN	x			

RB1	x			
ARID1A	x	x		
FOXA1	x			
NCOR1	x			
SPEN	x			
BYSL	x			
CBFB	x			
CTCF	x			
MAP2K4	x			
MED23	x			
RUNX1	x			
TBX3	x			
B2M	x			
GPD2	x			
HMCN2	x			
KDM6A	x			
MBD3L3	x			
PLK2	x			
PLIN4	x			
NBPF12	x			
LAMA5			x	
LAMC3			x	
COL6A5			x	
MUC12			x	
MAP2K3			x	
KDR				x
APC				x
ATM				x
FGFR3				x

Table S6: Studies excluded from analysis

Study	Reason for exclusion
Menon et al., 2023	Abstract only; lack of full text article
El Ansari et al., 2022	Copy number alteration on single gene
Elagali et al., 2021	Single gene analysis
Cornen et al., 2014	Not African population specific
Suspitsin et al., 2014	Not African population specific
Tomasetti et al, 2017	Not African population specific
Friebel et al., 2019	Germline focused; single gene analysis
Lee K et al., 2019	Not African population specific
Hamdi Y et al., 2021	Single gene analysis
Berger et al., 2019	Not African population specific

Table S7: PRISMA 2020 Checklist

Section and Topic	Item #	Checklist item	Location where item is reported
**TITLE**			
Title	1	Identify the report as a systematic review.	Pg 1
**ABSTRACT**			
Abstract	2	See the PRISMA 2020 for Abstracts checklist.	Pg 1
**INTRODUCTION**			
Rationale	3	Describe the rationale for the review in the context of existing knowledge.	Pg. 2-3
Objectives	4	Provide an explicit statement of the objective(s) or question(s) the review addresses.	Pg. 3
**METHODS**			
Eligibility criteria	5	Specify the inclusion and exclusion criteria for the review and how studies were grouped for the syntheses.	Pg. 15
Information sources	6	Specify all databases, registers, websites, organisations, reference lists and other sources searched or consulted to identify studies. Specify the date when each source was last searched or consulted.	Pg. 15
Search strategy	7	Present the full search strategies for all databases, registers and websites, including any filters and limits used.	Pg.15-16, Tables S1,2,3,4
Selection process	8	Specify the methods used to decide whether a study met the inclusion criteria of the review, including how many reviewers screened each record and each report retrieved, whether they worked independently, and if applicable, details of automation tools used in the process.	Pg.15-16,18
Data collection process	9	Specify the methods used to collect data from reports, including how many reviewers collected data from each report, whether they worked independently, any processes for obtaining or confirming data from study investigators, and if applicable, details of automation tools used in the process.	Pg.16-17,18
Data items	10a	List and define all outcomes for which data were sought. Specify whether all results that were compatible with each outcome domain in each study were sought (e.g. for all measures, time points, analyses), and if not, the methods used to decide which results to collect.	Pg. 17
	10b	List and define all other variables for which data were sought (e.g. participant and intervention characteristics, funding sources). Describe any assumptions made about any missing or unclear information.	Pg. 17
Study risk of bias assessment	11	Specify the methods used to assess risk of bias in the included studies, including details of the tool(s) used, how many reviewers assessed each study and whether they worked independently, and if applicable, details of automation tools used in the process.	Pg. 17
Effect measures	12	Specify for each outcome the effect measure(s) (e.g. risk ratio, mean difference) used in the synthesis or presentation of results.	Pg. 18
Synthesis methods	13a	Describe the processes used to decide which studies were eligible for each synthesis (e.g. tabulating the study intervention characteristics and comparing against the planned groups for each synthesis (item #5)).	Pg. 17
	13b	Describe any methods required to prepare the data for presentation or	Pg. 17-

Section and Topic	Item #	Checklist item	Location where item is reported
		synthesis, such as handling of missing summary statistics, or data conversions.	18
	13c	Describe any methods used to tabulate or visually display results of individual studies and syntheses.	Pg. 17
	13d	Describe any methods used to synthesize results and provide a rationale for the choice(s). If meta-analysis was performed, describe the model(s), method(s) to identify the presence and extent of statistical heterogeneity, and software package(s) used.	Pg. 18
	13e	Describe any methods used to explore possible causes of heterogeneity among study results (e.g. subgroup analysis, meta-regression).	N/A[1]
	13f	Describe any sensitivity analyses conducted to assess robustness of the synthesized results.	N/A[1]
Reporting bias assessment	14	Describe any methods used to assess risk of bias due to missing results in a synthesis (arising from reporting biases).	Pg. 17
Certainty assessment	15	Describe any methods used to assess certainty (or confidence) in the body of evidence for an outcome.	N/A[1]
**RESULTS**			
Study selection	16a	Describe the results of the search and selection process, from the number of records identified in the search to the number of studies included in the review, ideally using a flow diagram.	Pg. 4-5, Figure 1
	16b	Cite studies that might appear to meet the inclusion criteria, but which were excluded, and explain why they were excluded.	Table S6
Study characteristics	17	Cite each included study and present its characteristics.	Table 1
Risk of bias in studies	18	Present assessments of risk of bias for each included study.	Table 2
Results of individual studies	19	For all outcomes, present, for each study: (a) summary statistics for each group (where appropriate) and (b) an effect estimate and its precision (e.g. confidence/credible interval), ideally using structured tables or plots.	Table 1and Figures 5,6
Results of syntheses	20a	For each synthesis, briefly summarise the characteristics and risk of bias among contributing studies.	Pg. 4-5
	20b	Present results of all statistical syntheses conducted. If meta-analysis was done, present for each the summary estimate and its precision (e.g. confidence/credible interval) and measures of statistical heterogeneity. If comparing groups, describe the direction of the effect.	Pg. 6
	20c	Present results of all investigations of possible causes of heterogeneity among study results.	Pg. 6-8
	20d	Present results of all sensitivity analyses conducted to assess the robustness of the synthesized results.	N/A[1]
Reporting biases	21	Present assessments of risk of bias due to missing results (arising from reporting biases) for each synthesis assessed.	Table 2
Certainty of evidence	22	Present assessments of certainty (or confidence) in the body of evidence for each outcome assessed.	N/A[1]
**DISCUSSION**			
Discussion	23a	Provide a general interpretation of the results in the context of other evidence.	Pg. 10-12
	23b	Discuss any limitations of the evidence included in the review.	Pg. 12

Section and Topic	Item #	Checklist item	Location where item is reported
	23c	Discuss any limitations of the review processes used.	Pg. 13
	23d	Discuss implications of the results for practice, policy, and future research.	Pg. 14
**OTHER INFORMATION**			
Registration and protocol	24a	Provide registration information for the review, including register name and registration number, or state that the review was not registered.	Pg. 15
	24b	Indicate where the review protocol can be accessed, or state that a protocol was not prepared.	Pg. 15
	24c	Describe and explain any amendments to information provided at registration or in the protocol.	Pg. 4
Support	25	Describe sources of financial or non-financial support for the review, and the role of the funders or sponsors in the review.	Pg. 18
Competing interests	26	Declare any competing interests of review authors.	Pg. 18
Availability of data, code and other materials	27	Report which of the following are publicly available and where they can be found: template data collection forms; data extracted from included studies; data used for all analyses; analytic code; any other materials used in the review.	Data available on request

[1] Not a meta-analysis; no pooling synthesis was performed.

From: Page MJ, McKenzie JE, Bossuyt PM, Boutron I, Hoffmann TC, Mulrow CD, et al. The PRISMA 2020 statement: an updated guideline for reporting systematic reviews. BMJ 2021;372:n71. doi: 10.1136/bmj.n71. This work is licensed under CC BY 4.0. To view a copy of this license, visit https://creativecommons.org/licenses/by/4.0/ .
For more information, visit: http://www.prisma-statement.org/
